# Collagen Type III Metabolism Evaluation in Patients with Malignant Head and Neck Cancer Treated with Radiotherapy

**DOI:** 10.1155/2018/8702605

**Published:** 2018-03-26

**Authors:** Klaudia Mazurek, Krzysztof Siemianowicz, Wirginia Likus, Ewa Pierzchała, Robert Kwiatkowski, Jarosław Markowski

**Affiliations:** ^1^Department of Aesthetic Medicine, School of Pharmacy with the Division of Laboratory Medicine in Sosnowiec, Medical University of Silesia, Katowice, Poland; ^2^Department of Biochemistry, School of Medicine in Katowice, Medical University of Silesia, Katowice, Poland; ^3^Department of Anatomy, School of Health Sciences in Katowice, Medical University of Silesia, Katowice, Poland; ^4^Katowice Oncology Center, Radiotherapy Department, Katowice, Poland; ^5^Department of Laryngology, School of Medicine in Katowice, Medical University of Silesia, Katowice, Poland

## Abstract

Ionizing radiation affects the metabolism of key proteins of extracellular matrix including type III collagen, an important component of human skin. The aim of the work is an analysis of the impact of radical and palliative radiotherapy on collagen type III synthesis in patients with head and neck cancer. The test group consisted of 56 males with histopathologically confirmed head and neck cancer, for whom radiotherapy was applied as a form of radical or palliative treatment. The level of procollagen III aminoterminal propeptide (PIIINP), which is a marker of collagen type III synthesis, was determined in blood serum before radiotherapy, immediately following radiotherapy, and 3 months after it was finished. As a result of radical radiotherapy a statistically significant decrease of PIIINP levels in serum (*p* < 0.0001) was observed, both immediately after the radiotherapy and 3 months after the end of the treatment. Also the palliative radiotherapy caused a significant decrease of PIIINP right after the treatment (*p* = 0.0052), as well as during the examination performed 3 months later (*p* = 0.0004). The achieved results suggest that PIIINP can be used as a marker helpful in assessing radiation damage to connective tissue.

## 1. Introduction

Radiotherapy (RTH) is one of the most commonly applied treatments for malignant tumors. It is defined as a local therapy, which uses ionizing radiation to destroy cancer cells. Radiotherapy constitutes an important treatment aspect, not only in the case of malignant tumors of the head and neck. It is successfully used in treating tumors located elsewhere.

The following division may be applied, taking into account the therapeutic effect of radiotherapy:

- Radical radiotherapy: the aim of the treatment is a full recovery of a patient [1].
- Palliative radiotherapy: the therapy is applied in the case of a significant advancement of a disease and its main aim is a local seizure of tumor and maximal lengthening of a patient's life, or in extreme cases, improving the comfort of a patient's life, mainly by minimizing pain [1, 2].

Ionizing radiation's main impact is placed on structures described as biological cellular shields (biological membranes and DNA), by destroying them indirectly and directly. As a consequence of photon absorption, the center is ionized and electrons detach, significantly damaging the most sensitive cell elements. Such a mechanism of damage to living matter is defined as direct. Definitely more types of cytological radiation damage occur in the indirect mechanism, depending on the reactive oxygen species (ROS) generated in the water radiolysis process [3].

During radiotherapy, in addition to cancer cells, normal epithelial cells and connective tissue cells located in the radiated area surrounding the tumor are also affected by strong destructive mechanisms [4]. Damage to normal tissue is defined as radiation-induced reaction. Radiation dermatitis is estimated to affect 80–95% of patients undergoing radiotherapy [5, 6], while the head and neck region, next to the breast and perineal area, is particularly prone to radiation-induced skin reactions [7]. As a result of the treatment with ionizing radiation of head and neck cancers, damage is done to the skin of the face, neck, and décolleté, which are areas generally conditioning the aesthetics.

The first visible reaction of the skin exposed to ionizing radiation is erythema. Along with the successive absorbed fractions, the dryness associated with the fine-scaling “dry” exfoliation of epidermis increases and is followed by wet exfoliation. The swelling becomes visible and, over time, also epilation and pigmentation disorders [4, 8].

Damage is not only done to the epidermis but also to the connective tissue. Subsequent doses of ionizing radiation, absorbed through the skin of the patient during treatment, cause progressive destruction of parts of dermis cells. The remaining, preserved fibroblast pool is overactivated, resulting in an increased synthesis of collagen types I and III, irregularly arranged, as manifested in the form of fibrosis foci. Telangiectasia is also characteristic for late radiation reactions caused by damage to vascular endothelium [9, 10]. A particular intensification of radiation-induced skin reactions is recorded for radiotherapy combined with chemotherapy.

The assessment of the level of collagen type III synthesis is extremely interesting in the context of postradiation fibrosis of the skin. This collagen is a structural protein of the extracellular matrix, which is characteristic for soft tissue. It constitutes 10–15% of skin collagen [9]. Collagen biosynthesis is a complex, multistage process. The resulting macromolecule of procollagen is transported to the Golgi apparatus and then secreted beyond the cell. In the extracellular space, the extensive, aminoterminal (N-terminal), and carboxy-terminal (C-terminal) peptides, which are specific markers of protein synthesis, are cut off. The cutting off of the propeptides transforms procollagen into tropocollagen. Subsequent tropocollagen molecules aggregate, eventually forming collagen fiber [11, 12]. As a consequence of the biosynthesis of collagen type III, the procollagen III aminoterminal propeptide (PIIINP) is released, and its marking in the patient's blood serum determines the intensity of the process of synthesis of this type of collagen. In addition to the transforming growth factor *β*, the PIIINP is a recognized biomarker for fibrosis [10].

The aim of the study is to analyze the effects of radical and palliative radiotherapy on collagen type III synthesis in patients with diagnosed head and neck cancer.

## 2. Material and Methods

### 2.1. The Study Group

The test group was composed of 56 men aged 39–85 (average age: 62.9 ± 9.3 years) with histopathologically confirmed head and neck malignant tumor with the applied radical or palliative treatment. Patients treated with radical radiotherapy have not been presenting distant metastases (T1N0M0-T4aN1M0); however in the palliative therapy group cancer was much more advanced (T3N2M0-T4bN3M1).

Patients who were qualified to participate in the research project were informed in advance about all stages and procedures involved and agreed to participate in the research.

The research started in July 2014 and was completed in January 2016. The research protocol was approved by the Bioethics Committee of the Medical University of Silesia in Katowice, Poland (Resolution number KNW/0022/KB1/16/I/14 dated 22.04.2014).

*Criteria of Inclusion into the Research Group*

- Male.
- Histopathologically confirmed malignant tumor of the head and neck.
- Application of radical or palliative radiotherapy.

*Exclusion Criteria*

- Chemotherapy.
- Autoimmune diseases of connective tissue.
- Diabetes.
- Renal failure.
- Thyroid and adrenal disease.
- Glucocorticoid therapy.
- Malnutrition.
- Dermatoses that may affect the level of markers of connective tissue remodeling.

The patients participating in the research projects were divided into two subgroups.

*I Group: Patients Qualified for Radical Treatment*

- Group size: 28 people.
- Patients' age: 43–85 (average age: 64.0 ± 10.4 years).
- Average time of radiotherapy: 6 weeks.
- Total amount of radiation received during treatment: 60–66 Gy.
- Number of fractions: 30–33.
- Fraction dose: 2 Gy.
- Energy of the applied ionized radiation: 6 MeV.

*II Group: Patients Qualified for Palliative Treatment*

- Group size: 28 people.
- Patients' age: 39–78 (average age 61.9 ± 8.2 years).
- Average time of radiotherapy: 1 week.
- Total amount of radiation received during treatment: 20 Gy.
- Number of fractions: 5.
- Fraction dose: 4 Gy.
- Energy of the applied ionized radiation: 6 MeV.

Highly specialized techniques were applied in radiating the patients, such as the IMRT— intensity modulated radiation therapy or the VMAT—volumetric modulated arc therapy.

### 2.2. Tests Materials

Test materials consisted of samples of patients' blood taken in the vacuum system from the ulnar vain, according to the established scheme:

 
*T*_0_: taken immediately before the radiotherapy (sample I).
 
*T*_1_: taken immediately after the end of the radiotherapy cycle (sample II).
 
*T*_2_: taken after three months following the end of the therapy (sample III).

After centrifugation, adequately protected serum was stored in temperature below −70°C until the marking was performed.

### 2.3. Methodology of Marking

The concentration of the collagen III synthesis marker (PIIINP) was determined by radioimmunoassay (RIA). Commercial UniQ® PIIINP RIA kits were used (manufacturer: Orion Diagnostica Oy, Espoo, Finland).

### 2.4. Statistical Methods

The analysis included results from patients, where a set of test material (i.e., all three samples) was obtained. The arithmetic means and standard deviations were calculated for the indicated parameters. The Shapiro-Wilk test was applied to verify normal distribution, showing a significant deviation from the normal distribution. In this case, it was decided that a further statistical analysis of nonparametric significance tests would be applied (Friedman's test with post hoc tests, which included the Bonferroni's correction).

The following levels of statistical significance were adopted:

- *p* > 0.05: not significant (NS).
- *p* < 0.05: statistically significant.
- *p* < 0.01: high statistical significance.
- *p* < 0.001: very high statistical significance.

All statistical analyses were performed using Statistica and Microsoft Office Excel software.

## 3. Results

### 3.1. The PIIINP Level in Blood Serum in Patients Treated with Radical Radiotherapy

There was a statistically significant decrease in the concentration of the collagen III synthesis marker directly after treatment (*T*_1_: 4.19 ± 3.42 *μ*g/L) versus the pretreatment value (*T*_0_: 8.67 ± 2.9 *μ*g/l). The change in PIIINP level is characterized by very high statistical significance (*p* < 0.0001).

An analogous situation is seen with respect to time points* T*_0_ and* T*_2_ (*p* < 0.0001). PIIINP level measured in serum of patients 3 months following the treatment (*T*_2_: 4.64 ± 2.7 *μ*g/L) is significantly lower than the level settled before treatment (*T*_0_: 8.67 ± 2.9 *μ*g/l).

**Figure 1:**
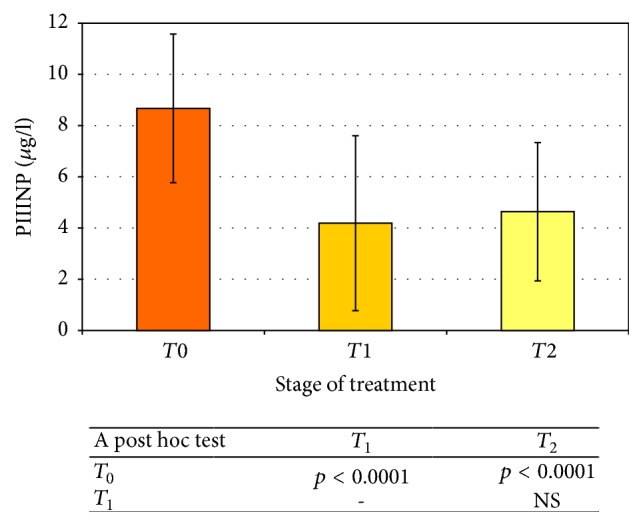
The PIIINP concentration before (*T*_0_) and after (*T*_1_,* T*_2_) radical radiotherapy (average ± SD). The statistical significance of differences (or lack of it) is shown in the table below the chart.

A comparison of serum levels taken immediately after treatment (*T*_1_) with the 3-month posttreatment (*T*_2_) level showed no statistically significant difference. Changes in the PIIINP level in serums of patients undergoing radical radiotherapy are shown in Figure 1.

### 3.2. The PIIINP Level in Blood Serum in Patients Treated with Palliative Radiotherapy

There was a statistically significant (*p* = 0.0052) decrease in the concentration of the collagen III synthesis marker immediately after treatment (*T*_1_: 7.3 ± 3.2 *μ*g/l) with respect to the preradiotherapy value (*T*_0_: 10.0 ± 4, 5 *μ*g/l). The 3-month posttreatment (*T*_2_: 5.0 ± 2.0 *μ*g/l) markers showed a further significant decrease in PIIINP compared with the value obtained before the start (*T*_0_: 10.0 ± 4.5 *μ*g/l). The observed relationship shows a very high statistical significance (*p* = 0.0004).

**Figure 2:**
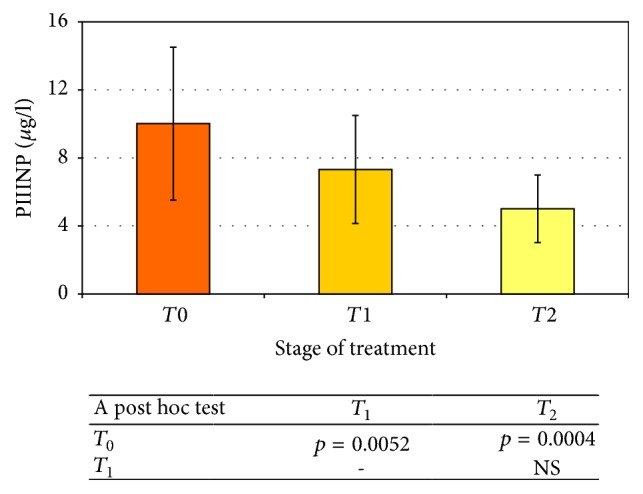
The PIIINP concentration before (*T*_0_) and after (*T*_1_,* T*_2_) palliative radiotherapy (average ± SD). The statistical significance of differences (or lack of it) is shown in the table below the chart.

The difference between PIIINP concentrations during* T*_1_ and* T*_2_ was not statistically significant. The changes in PIIINP level in the serum of patients undergoing palliative radiotherapy are shown in Figure 2.

## 4. Discussion

When analyzing biological and molecular effects of radiotherapy on living matter, the impact of ionizing radiation on the connective tissue seems to be particularly important, primarily due to its prevalence and the complexity of its functions. The characteristic feature of this tissue is its constant remodeling, which consists of continuous processes of biosynthesis and degradation of components of extracellular matrix. One of the key components of ECM is collagen, an extremely important component of skin, which accounts for nearly 75% of its dry mass. Apart from the skin, the discussed protein is identified in the structures of tendons, blood vessels, bones, dentins, ligaments, and cartilage [13]. Among all the collagens, collagen type III, next to collagen type I, plays a key role in human skin [14]. It therefore seems appropriate to evaluate the effect of radiotherapy on its biosynthesis. The skin is referred to as one of the most radiation sensitive organs [5].

As the results of our research show, radiotherapy, both radical and palliative, significantly alters the remodeling of collagen type III. It causes a significant decrease in the marker of its synthesis in the blood serum of patients, as a result of a decreased collagen type III synthesis.

Only a few studies on similar issues that analyze changes in the synthesis of collagen following radiotherapy can be found in available literature. Keskikuru et al. [15] observed breast cancer patients who underwent surgery, followed by adjuvant radiotherapy. The test material was suction blister fluid (SBF) obtained by a suction chamber which, through the generated vacuum, induces blistering, from which the test material is collected. The locally acquired PIIINP level in SBF taken from the treated breast prior to the initiation of RTH was significantly lower than the posttreatment level. The maximum value of propeptide was recorded one month after the end of therapy. An increased local marker level was observed within the next two years after radiotherapy.

The results presented by Keskikuru et al. are significantly different from our results, in which patients with both radical and palliative radiotherapy had a statistically significant decrease in the PIIINP at the end of the treatment, compared to baseline values. Also, the level of propeptide determined in the test performed 3 months after treatment was significantly lower than the pretreatment markers. The results obtained by the abovementioned Finnish researchers refer to local changes in PIIINP levels induced by ionizing radiation.

Therefore, they may not constitute a direct reference to the results of this study. In addition, the total dose of radiation applied, the type and location of the tumor, and the gender of the patients were different in both studies.

Riekki et al. [9] conducted observations with the application of methodology similar to Keskikuru, on a group of women with breast cancer who underwent follow-up radiotherapy. Patients were measured for PIIINP levels using noninvasive SBF methods. A significantly higher level of PIIINP was revealed in SBF extracted from areas treated with ionizing radiation compared to those indicated in nonirradiated skin material. The results suggest that the fibrosis process is especially evident in late postradiation reactions. The mechanism of postradiation fibrosis has not yet been precisely defined [9]. An important role in its formation is attributed to the transforming growth factor *β* (TGF-*β*), a cytokine synthesized by platelets, macrophages, lymphocytes, and neutrophils, significantly promoting collagen biosynthesis and thus inducing the PIIINP release [10]. The pathomechanism of fibrosis also most likely involves the mast cells of the skin. Cytokines released by mastocytes are participants of fibrosis [9].

A comparative analysis of our findings with the results of Finnish researchers shows that the local level of collagen III remodeling marker is substantially different from marker values found in the patients' serum. No studies have been found in literature on propeptide level changes after radiotherapy in patients with head and neck cancer treated with radiotherapy.

The PIIINP is a marker of collagen type III synthesis that can be used for the diagnosis and monitoring of other conditions than radiation-induced skin reactions. An attempt to use it in evaluating collagen remodeling following radiotherapy appears to be still a pioneering idea.

Aminoterminal propeptide of procollagen type III may be used as an objective diagnostic tool for pulmonary fibrosis in systemic sclerosis [16] or in the monitoring of heart fibrosis [10]. A significantly higher level of propeptide is also registered in patients with fibrosis in liver structures [17]. The PIIINP also appears to be a useful diagnostic tool in assessing the pathogenesis of hypertrophic scars resulting from abnormal fibroblast proliferation and overproduction of ECM structural proteins. The recorded significant increase in collagen I and III synthesis correlates with an increased TGF-*β* activity [18]. Propeptide is an important marker in patients with heart failure with preserved ejection fraction (HFPEF) or hypertrophic cardiomyopathy (HCM) characterized by unexplained left ventricular hypertrophy and the presence of fibrosis as an effect of increased synthesis of collagen types I and III [19, 20]. It is treated as a prognostic indicator in the course of cancer. Santala et al. [21] proposed the use of the PIIINP in patients with ovarian cancer in whom they observed its elevated levels correlating with the advancement of the disease. Wiklund et al. [22], on the other hand, used the propeptide as one of the connective tissue remodeling markers in patients with bone sarcoma.

To date, many systems have been developed to assess the severity of early and long-term skin radiation-induced skin reactions, including RTOG/EORTC (Radiation Therapy Oncology Group/European Orgnization for Research and Treatment) or NCI-CTC (National Cancer Institute-Common Toxicity Criteria) [23]. The created systems are primarily based on visual classification of damage in the epidermis and mucous membranes performed by a specialist and a subjective assessment of the patient. There are still no diagnostic methods for assessing radiation-induced damage to the skin.

Analyzing the results of our research as well as referring to results achieved by other researchers, as mentioned above, it can be stated that PIIINP can be considered as a diagnostic marker helpful in assessment of radiation damage of one of the most crucial structural protein of human dermis.

## 5. Conclusions

The achieved results indicate that type III collagen biosynthesis in patients with head and neck cancer treated with radiotherapy is significantly decreased both in patients receiving higher (60–66 Gy) and lower (20 Gy) total radiation dose. Radiotherapy, both radical and palliative, causes statistically significant decrease of PIIINP concentration in blood serum. Decreased level of collagen type III synthesis marker is noticeable immediately after the therapy as well as 3 months after the finished treatment in both studied groups. As a result of the treatment, the decrease of collagen synthesis which creates connective tissue scaffold of skin seems to have also a significant impact on pathogenesis of early radiation reactions.

Procollagen III aminoterminal propeptide not only is a marker that is more and more widely used nowadays in the assessment of cancer, but is also an important diagnostic tool in the pathogenesis of fibrosis.

The results presented in this paper indicate that PIIINP can also be used as an indicator of radiation-induced damage to the connective tissue.
